# Subdiaphragmatic Renal Ectopia: Case Report and Review of the Literature

**DOI:** 10.1155/2016/1084917

**Published:** 2016-09-07

**Authors:** Eleftherios Zolotas, Rajesh G. Krishnan

**Affiliations:** Children's Kidney Centre, University Hospital of Wales, Cardiff, UK

## Abstract

*Background*. We report the case of a male infant whose right kidney migrated to an ectopic position after birth. The migration of a kidney in postnatal life without any symptoms has not been reported in literature so far.* Case Presentation*. In a series of antenatal and the first postnatal ultrasound scans, the right kidney was normally located within the right renal fossa. During the first 3 months of life, the kidney migrated to a subdiaphragmatic position. This was confirmed on MRI scan. The infant was asymptomatic with normal renal function and blood pressure.* Conclusion*. Postnatal migration of a kidney has been described in cases of diaphragmatic hernia or nephroptosis. In this report, we describe a case of kidney migration where there were no underlying anatomical defects to provide an explanation for the kidney migration. This is the first report in literature of a case of postnatal migration of a kidney.

## 1. Background

During organogenesis, the kidneys ascend to their normal position between the 6th and the 9th week of gestation [1]. Any disturbance of this process can lead to renal ectopia [2]. The event of the migration of a kidney in postnatal life is rare. It is usually associated with congenital diaphragmatic hernia [3–6] or trauma [7]. In nephroptosis, the affected kidney is mobile and typically descends in the upright position because of a defect in the renal fascia and the perirenal fat [8].

We report the case of a male infant whose right kidney migrated from the renal fossa and assumed an ectopic position after birth. The kidney migration could not be explained by any of the known causes.

## 2. Case Presentation

A Caucasian, 27-year-old female was being followed up during her second pregnancy. Her past medical and obstetric history was unremarkable. This was her second pregnancy; her older child from the first pregnancy was well. There was no family history of renal diseases or congenital abnormalities. She was not on treatment with any medications. A dating ultrasound scan was performed at 15 weeks of gestation showing a single live foetus.

The pregnancy advanced uneventfully. The anomaly ultrasound scan at 20^+2^ weeks of gestation showed evidence of duplex right kidney with dilatation of the upper moiety. The ultrasound scan was repeated at 23^+6^ weeks of gestation with the same findings. The dilatation of the upper moiety was measured at 10.8 mm. The right kidney was in normal position and there was satisfactory parenchymal differentiation. The left kidney, the ureters, and the bladder appeared normal. The amniotic fluid index was normal throughout the pregnancy. A follow-up scan at 32^+1^ weeks of gestation showed the right duplex kidney with resolution of the dilatation previously seen. The foetal growth was optimal. No other abnormalities were detected in the antenatal scans.

**Figure 1:**
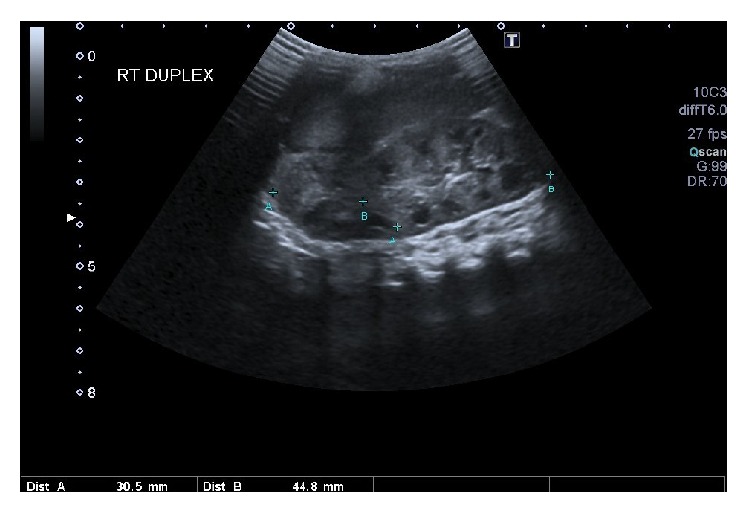
Ultrasonography of the right kidney at 12 days of age showing evidence of duplex system. The kidney was visualised within the right renal fossa.

She delivered a male infant via normal vaginal delivery at 39^+2^ weeks of gestation. The infant was born in good condition without any perinatal complications. An ultrasound scan (Figure 1) was performed at 12 days of age. This scan showed features consistent with an uncomplicated right duplex kidney measuring 6.1 cm. The left kidney had a single collecting system and was measuring 5.2 cm. Both kidneys were visualised within the renal fossae and had normal parenchymal differentiation. There was no pelvic dilatation and the ureters were not visible in either of the kidneys. The bladder looked normal. Clinically, the infant was asymptomatic with normal blood pressure and renal function. There were no other evident congenital abnormalities.

**Figure 2:**
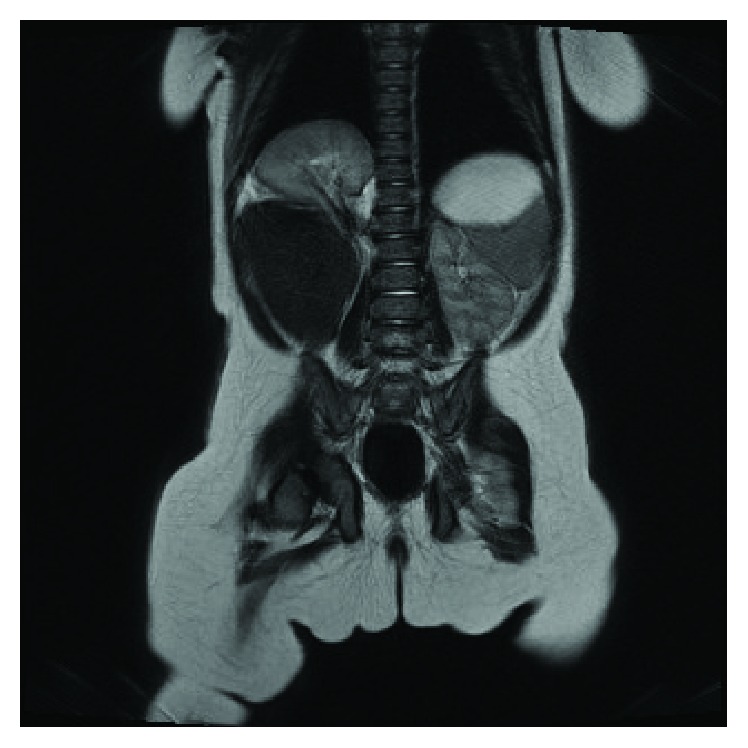
MRI coronal view of the abdomen and the chest. The right kidney appears in a subdiaphragmatic position.

The infant had a follow-up ultrasound scan at 3.5 months of age. During that scan, it was difficult to visualise the right kidney which was not within the renal fossa but in a higher position behind the liver. MRI (Figure 2) was performed at 4.5 months of age to determine the locus of the right kidney and possible associated abnormalities. It became evident that the right kidney was lying posteriorly and superiorly to the liver, below the diaphragm. There was no suggestion of duplex system and no features consistent with herniation were demonstrated. The size and the differentiation of both kidneys were normal. The diaphragm was intact and apart from the movement of the right kidney, there were no other abnormalities seen. There was no history of trauma.

**Figure 3:**
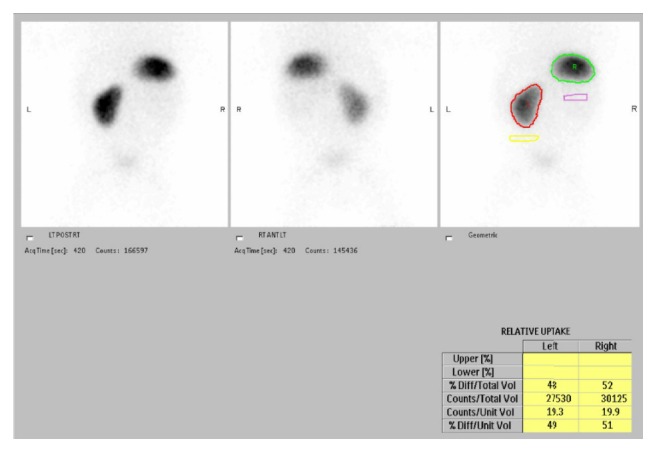
DMSA scan showing the absence of renal scarring and normal differential function.

A DMSA scan (Figure 3) was performed at 6.5 months of age which showed normal differential renal function; 52% on the right kidney and 48% on the left kidney were without any evidence of scarring. There was equal function in the upper and the lower poles of both kidneys with no evidence of duplex system. The infant had normal renal function and blood pressure and grew along his centile lines.

## 3. Discussion

In human foetus, the development of the kidney proceeds through a series of successive phases, each characterised by the development of a more advanced kidney: the pronephros, mesonephros, and metanephros. The latter is the most mature form of foetal kidney and persists as the definitive kidney [1].

The metanephros is formed during the 5th week of gestation as a result of the interaction between the ureteric bud and the metanephric blastema at the level of the 1st sacral vertebra. Between the 6th and the 9th week of gestation, the kidneys ascend to the level of the 12th thoracic vertebra under the suprarenal glands. The mechanism that leads to the ascent of the kidneys is not an active migration but rather the result of the differing growths of the sacral and lumbar regions. The renal fascia (Gerota's fascia) envelopes the kidney and the suprarenal gland and along with the perirenal fat supports it and keeps it within the renal fossa [1].

In renal ectopia, the kidney lies outside the renal fossa as a result of disordered or absent ascent during organogenesis. The site of an ectopic kidney is either deep (usually pelvic but also lumbar or iliac) or less commonly high (intrathoracic or subdiaphragmatic). In some cases, the ectopic kidney crosses the midline and lies on the same side with the other kidney (crossed ectopia) [2]. Renal ectopia is not uncommon (1–5 per 1,000) and most cases are asymptomatic. It is a congenital condition and occurs early during organogenesis (6th–9th weeks).

However, high renal ectopia is a rare condition (one in 22 cases of ectopic kidneys) [9]. It occurs four times more frequently in men and it is found on the left side twice as often as on the right. It can present with ipsilateral hypochondrial pain and can be accompanied by vesicoureteric reflux [10, 11]. In asymptomatic cases, it can be an incidental finding when imaging is performed for other reasons [12–14]. In the absence of trauma or anatomical defects, a high renal ectopia is presumed to be a congenital abnormality.

Postnatal migration of a kidney is a rare occurrence. It has been reported in cases of congenital diaphragmatic hernia in which the kidney can herniate into the thorax. It is typically a symptomatic condition with neonatal presentation that requires surgical intervention [3–6]. Acquired herniation of the kidney through the diaphragm has also been reported posttraumatically [7]. However, in our case, there was no evidence of herniation both radiologically and clinically. There was no history of trauma as well.

Mobile kidney is a feature of nephroptosis in which the kidney descends during a position change from supine to upright, possibly due to a defect in the renal fascia and perirenal fat. It is a fairly rare condition and it is commonly manifested with colicky pain [8]. Management of symptomatic cases entails surgical intervention. Robotic laparoscopic nephropexy can be an alternative to open surgery and it has been already successfully attempted in a child [15]. Nephroptosis is obviously not relevant to the kidney migration in our case.

The ultrasonographic finding of a duplex kidney was not confirmed with the MRI. Ultrasonography shows high specificity and low sensitivity for duplex kidneys. However, false positive results can still occur. False positives may represent either other congenital abnormalities or entirely normal kidneys [16, 17]. There is evidence that MRI is superior to ultrasonography for the diagnosis of duplex kidneys in children [18].

In this case, we report the right kidney migrating from a normal to a subdiaphragmatic position during the first 3 months of life. This was confirmed in a series of ultrasound and MRI scans. There has been no similar case described previously.
